# The role of complement immune response on artemisinin-based combination therapy in a population from malaria endemic region of Western Kenya

**DOI:** 10.1186/s12936-020-03242-4

**Published:** 2020-04-29

**Authors:** Christine N. L. Wanjala, Elke Bergmann-Leitner, Hoseah M. Akala, Geoffrey Odhiambo, Bernhards R. Ogutu, Ben Andagalu, Edwin Kamau, Daniel Ochiel

**Affiliations:** 1 Department of Emerging and Infectious Diseases (DEID), United States Army Medical Research Directorate-Africa (USAMRD-A), Kenya Medical Research Institute (KEMRI)/Walter Reed Project (WRP), Kisumu, Kenya; 2 Malaria Biologics Branch, Walter Reed Army Institute of Research, Silver Spring, MD USA; 3 U.S. Military HIV Research Program, Walter Reed Army Institute of Research, Silver Spring, MD USA; 4 School of Physical and Biological Sciences Zoology Department, Maseno University, Maseno, Kenya; 5 KEMRI, Nairobi, Kenya

**Keywords:** Malaria antibody, Complement, Malaria immunity, Artemisinin combination therapy, Western Kenya, Malaria holoendemic areas

## Abstract

**Background:**

Naturally acquired immunity (NAI), which is characterized by protection against overt clinical disease and high parasitaemia, is acquired with age and transmission intensity. The role of NAI on the efficacy of anti-malarial drugs, including artemisinin-based combinations used as the first-line treatment for uncomplicated *Plasmodium falciparum,* has not been fully demonstrated. This study investigated the role of NAI in response to artemisinin-based combination therapy (ACT), in symptomatic patients living in western Kenya, a high malaria transmission area.

**Methods:**

Sera samples from malaria immune participants (n = 105) in a therapeutic efficacy study were assessed for in vitro growth inhibitory activity against the 3D7 strain of *P. falciparum* using a fluorescent-based growth inhibition assay (GIA). Participants’ age and parasite clearance parameters were used in the analysis. Pooled sera from malaria naïve participants (n = 6) with no *Plasmodium* infection from malaria non-endemic regions of Kenya was used as negative control.

**Results:**

The key observations of the study were as follows: (1) Sera with intact complement displayed higher GIA activity at lower (1%) serum dilutions (*p *< 0.0001); (2) there was significant relationship between GIA activity, parasite clearance rate (*p *= 0.05) and slope half-life (*p *= 0.025); and (3) age was a confounding factor when comparing the GIA activity with parasite clearance kinetics.

**Conclusion:**

This study demonstrates for the first time there is synergy of complement, pre-existing immunity, and drug treatment in younger patients with symptomatic malaria in a high-transmission area.

## Background

People living in malaria endemic areas acquire natural immunity with age, with faster rates of acquisition occurring in high compared to low transmission settings [1]. Both humoral and cell-mediated immune responses are involved in generating effective immunity [2], with individuals who possess greater breadth of immunological responses being at a lower risk of developing overt clinical symptoms [3].

Naturally acquired immunity has been shown to influence malaria drug treatment outcomes by enhancing the efficacy of sub-optimal drug regimen and delaying the emergence of drug resistance, including the artemisinin-based combinations used as the first-line treatment of *Plasmodium falciparum* in most malaria endemic countries [4]. Artemisinin-based combination therapy (ACT) is still highly efficacious in sub-Saharan Africa (sSA) [5], but as malaria transmission reduces, and malaria immunity at population level wanes [6], the efficacy of ACT is likely to be impacted. It is, therefore, important to monitor and assess the effect of pre-existing acquired immunity on the efficacy of ACT as part of therapeutic efficacy studies (TESs).

Complement factors have been shown to play a key role in antibody-mediated immunity to malaria in humans [7]. Studies from malaria endemic areas have demonstrated that *P. falciparum* exposure is associated with growth-inhibitory activity in total [8], and merozoite surface protein 1 (MSP-1) or apical membrane antigen 1 (AMA-1)-specific IgG fractions [9, 10]. Growth inhibition assays (GIA) assesses the functional activity of sera to reduce/inhibit red blood cell (RBC) invasion and/or intra-RBC growth of *P. falciparum* in vitro [11]. Using GIA, the present study aimed to assess the role of complement and pre-existing immunity in the response to malaria drug treatment in symptomatic patients living in a malaria high-transmission area of western Kenya.

## Methods

### Study site, population and sample collection

This is a sub-study that analysed samples from a TES conducted in Kombewa district hospital in Kisumu County, western Kenya from June 2013 through November 2014. Detailed clinical study will be reported elsewhere. Briefly, Kisumu County is a malaria holoendemic lake region with intense malaria transmission through-out the year, with annual entomological inoculation rates (EIR) of 31.1 infected bite per year [12]. This was a two-arm, randomized open-label study, where patients presenting with uncomplicated malaria at the Kombewa district hospital between the ages of 6 months to 65 years were recruited (Table 1). Study participants were randomized to receive artemether-lumefantrine (AL) or artesunate-mefloquine (ASMQ) using block randomization schemes with varying block sizes. Venous blood samples were collected at hours 0, 4, 8, 12, 18, 24 and then every 6 h until two consecutive smears became negative. Giemsa-stained films were prepared following World Health Organization (WHO) guidance and read by two independent expert microscopists. The geometric mean of the parasite count per microlitre from each participant at each sampling time point was then calculated. Participants were followed for a total of 42 days. A total of 118 participants were enrolled in the study, 59 from each arm. From these, 105 blood samples from study participants (46 participants who were < 5 years of age and 59 who were ≥ 5 years of age) herein regarded as immune sera sample were successfully analysed using the GIA.

**Table 1:** Demographic information for the study participants

	AL N = 59	ASMQ N = 59
Age–mean years (SD)	6.7 (7.1)	8.9 (10.1)
Age–median years (range)	5 (1–50)	6 (1–51)
Sex—female N (%)	34 (57.6%)	35 (59.3%)
Mean weight kg (SD)	24.0 (15.7)	25.0 (15.1)
Weight range	10.6–90.4	11.0–76.6
Temperature–mean °C (range)	38.0 (36.0–40.1)	37.5 (36.1–40.0)
Mean Hb g/dl (range)	10.6 (7.0–15.1)	10.8 (7.2–15.8)
Asexual parasite density—geometric mean per µL (95% CI)	38,759 (26,194–57,351)	32,789 (21,907–49,078)
Gametocyte carriage N (%)	51 (86.4%)	52 (88.1%)

Malaria episode was defined at a measured temperature of ≥ 37.5 °C with baseline parasitaemia of 2000–200,000 asexual parasites/µL. GIA activity was successfully assessed in 105 samples (52 AL arm and 53 ASMQ arm), where 46 (23 females) were < 5 years old, and 59 (38 females) were ≥ 5 years old

Pooled sera from six adult males 18–65 years old, who were *P. falciparum* negative, confirmed by microscopy and polymerase chain reaction (PCR), were used as non-immune control for the GIA experiments. These donors had blood groups A, B and O, and haemoglobin level of ≥ 13 g/dL, living in malaria non-endemic regions (Kericho and Nairobi), with no travel history to malaria endemic areas of Kenya in the last 6 months prior to blood donation. For the maintenance of the parasite culture and performing the GIA assays, red blood cells (RBCs) were obtained from blood group “O” donors, ages between 18 and 50 years, with haemoglobin level of 14–18 g/dL for males, and 12–16 g/dL for females living in a malaria non-endemic area (Kericho and Nairobi) with no travel history to malaria endemic areas in the last 6 months prior to donating blood. After collection, the blood was kept in cool boxes containing 2–8 °C ice parks with portable thermometers, which were safely transported to the central lab by courier service provider under monitored cold chain within 24 h. The cold chain, and sample integrity including absence of lysis and leakage was verified by the receiving technician and documented in the laboratory record book prior to processing as previously described [13]. Briefly, in 15 mL centrifuge tubes, 7 mL aliquots of the whole blood were added to 7 mL wash medium and then centrifuged at 800*g* for 10 min where most white blood cells (WBCs) in buffy coat were gently aspirated, followed by the entire supernatant, leaving the packed RBCs. The process was repeated three-time to remove all the remaining WBCs. The cells were then suspended in equivalent wash medium at 50% haematocrit (packed cells/wash medium v/v), then stored at 2–8 °C.

### Culture media

Culture medium used to maintain *P. falciparum* in vitro (Life technologies, Carlsbad, CA) was prepared as previously described [14]. Briefly, basic media comprised of 10.4 g RPMI powder (Invitrogen, Inc., Carlsbad, California, USA) combined with 2 g of glucose (Sigma Inc., St Louis, Missouri, USA) and 5.95 g HEPES (Sigma, USA) dissolved to homogeneity in 1 litre of de-ionized water and sterilized with a 0.2 µM filter. RPMI 1640 tissue culture media (TCM), for all parasite culture and drug dilutions, consisted of RPMI 1640 basic media with 0.5% Albumax II (Gibco, Grand Island, NY), 3.2% (vol/vol) sodium bicarbonate (Thermo Fisher Scientific Inc., Waltham, Massachusetts, USA) and 4 µg/mL hypoxanthine (Sigma Inc., St Louis, Missouri, USA). Complete RPMI 1640 media was stored at 2–8 °C. Serum free media consisted of 1.0 mL of 1.45 mM sterile hypoxanthine to 43.4 mL of RPMI basic medium, 1.6 mL of sterile sodium bicarbonate (Thermo Fisher Scientific Inc., Waltham, Massachusetts, USA), 7.5% and sterilized with 0.2 µM filter then put in 2–8 °C. 10% complete media for Control was prepared by adding 5 mL of serum to 43.4 mL of RPMI basic medium, 1.0 mL of 1.45 mM sterile hypoxanthine, 1.6 mL of sterile sodium bicarbonate, 7.5% then sterilized with 0.8 µM, 0.45 µM, 0.2 µM filters and put at 2–8 °C.

### *Plasmodium falciparum* culture

*Plasmodium falciparum* 3D7 strain, obtained through Biodefense and Emerging Infections Research Resources Repository (BEI Resources, NIAID, NIH) was maintained in continuous culture as previously described [13]. Briefly, *P*. *falciparum*-3D7 were suspended in 5 mL of complete media with serum (CMS) at 6% haematocrit in tissue culture flasks (Corning Glass Works, Corning, NY). The flasks were flushed with a gas mixture consisting of 5% oxygen, 5% carbon (IV) oxide and 90% nitrogen (Air Products Corp., Allentown, PA), sealed, and incubated at 37 °C. The medium was changed daily, and fresh RBCs added when required. Parasite growth was monitored microscopically by counting Giemsa-stained thin blood smears. When parasitaemia exceeded 3%, ring-stage parasites were synchronized by sorbitol lysis (5% d-sorbitol (Sigma, St. Louis, MO) as follows: Parasites were mixed and transferred into a 15 mL centrifuge tube and centrifuged at 800 g for 10 min. After removing the supernatant, 5% d-sorbitol pre-warmed to 37 °C was added to the RBC pellet, incubated for 10 min at 37 °C, vortexed for 1 min and re-centrifuged at 800 g for 10 min. After aspirating the supernatant and repeating the process twice using TCM, ≥ 90% ring forms was achieved.

### Serum preparation

Serum was obtained from blood samples as previously described [15] with minor modifications. Briefly, whole blood collected in plain (anticoagulant free) blood bags from each study participant was stored at 2–8 °C overnight to allow settling of the serum and cells portions. Serum portion was transferred into 50 mL centrifuge tubes, and centrifuged at 800 g for 10 min. The supernatant was transferred into 50 mL centrifuge tube to remove all cell components. This step was repeated three times, and the serum was stored at -65 to -80 °C until further use. The sera from the six male adults (non-immune) was pooled together forming approximately 2000 mL, which was used as negative control for subsequent experiments. Serum from each of the immune or non-immune pooled serum was divided into two equal portions. One portion was heat inactivated at 56 °C for 30 min in a water bath, as previously described [7]. Experiments were carried out in 1 or 10% sera, prepared as previously described [7]. Briefly, 10% immune or non-immune CMS was prepared by adding 0.5 mL participant’s serum to 4.5 mL serum free media. To prepare 1% immune or non-immune CMS, 0.5 mL of 10% immune/non-immune CMS was added to 4.5 mL serum free media, making a tenfold dilution. This was done for heat-inactivated and non-heat inactivated sera.

### Growth inhibition assay (GIA)

In vitro anti-plasmodial susceptibility assay was performed as previously described [14] with some modifications. Briefly, tenfold dilution in TCM was done for immune serum, non-immune serum, and negative control wells. Parasitaemia in the GIA assays of continuous culture that were greater than 3% were lowered to 1%, and adjusted to final 1–2% hematocrit. A ratio of 1:10 serum dilution in 100 µL final volume was dispensed on Nalgene Nunc, 96 flat-bottom well cell cultures sterile with lid microlitre plates (Magna, Leicestershire, UK), and 10 µL of 3D7 parasite was added in each well then incubate at 37 °C for 72 h in modular reservoirs, gassed with 90% nitrogen, 5% carbon dioxide and 5% oxygen. Parasite growth was evaluated using SYBR Green I fluorescence assay as follows: 4 µL of 10X SYBR Green I dye was added to 2 mL of malaria SYBR Green I lysis buffer [20 mM Tris (pH 7.5), 5 mM EDTA, 0.16% (wt/vol) saponin, and 1.6% (vol/vol) Triton X-100], and 100 µL of this mixture was transferred to each well. The plates were stored in the dark at ambient temperature for 24 h [16]. The plates were then examined for relative fluorescence units (RFUs) per well using a fluorescence plate reader (Tecan™ Morrisville, North Carolina, United States). Parasite replication was quantified for each serum sample (immune/pooled non-immune sera) and expressed as the mean percentage growth inhibition by:

$${\text{Percentage growth inhibition}} = \frac{{{\text{average RFUs in growth medium }} - {\text{average RFUs inpatients serum}}/{\text{pooled serum}}}}{{{\text{Average RFU in growth medium}}}} \times 100\%$$

Optimization was done to validate the assay and control reference ranges were established upon which the assay performance was monitored using anti-malarial drugs dihydroartemisinin (DHA) and lumefantrine (LU) that have known IC_50_ for the reference parasite strain. The drugs DHA and LU were used as positive control and tissue culture media (10% CMS) was used as negative control. All experiments were conducted in triplicate.

### Parasite clearance rates calculation

The statistical models used to estimate the parasite clearance measures and lag phase duration were fitted using the Parasite Clearance Estimator (PCE) tool developed by Worldwide Antimalarial Resistance Network (WWARN) [17]. Briefly, thick and thin smears of malaria blood slides were generated examined under light microscope at 100× magnification for presence of malaria parasites as part of monitoring treatment outcome. From the initial scan through slides with parasite counts/WBC count ratio less than 2 in thick smears were regarded as low density infection and reading done in this smear. The parasite density in this low density smears were estimated as the number of parasites, the asexual forms only, counted against the number of white blood cells (usually 200 for low density or 500 for extra low density with less than 10 parasites pre 200 WBCs). Slides with parasite counts/WBC count ratio exceeding two were counted in thin smear as infected RBC counts/total number of RBC counts (usually in at least 2000 RBCs). The readouts of counts per either WBCs or RBCs were converted to parasite densities at time points 0, 4, 8, 12, 18, 24, 30, 36, 42, and 48 h, then continued at least every 24 h until clearance depicted by until two subsequent negative malaria blood slides. A malaria blood film was considered negative if no parasite observed after scanning 200 high power fields (since a high quality HPF contains about 20 WBCs, this represents approximately 4000 WBCs). Data on parasite density was converted to csv file format and uploaded into the WWARN parasite clearance estimator.

Data was downloaded from the PCE and the following parameters were estimated: parasite clearance half-life, parasite clearance rate constant (K/h), and the estimated time to reduce parasitaemia by 50% (PC50), 90% (PC90), 95% (PC95), and 99% (PC99). Log transformed parasite density was plotted against time in hours to generate slope half-life (T_1/2_) which is defined as the time needed for parasitaemia to be reduced by half [18]. This constant is independent of starting value of parasitaemia. The slope half-life was calculated as follows:T_1/2_ = log_e_ (2)/K = 0.692/K, where K is the clearance rate constant. The clearance rate K represents the rate of parasite clearance after start of drug treatment.

### Statistical analysis

Data was analysed using GraphPad Prism version 5.03 (GraphPad software, Inc., San Diego, CA) and Minitab, Version 17 (Minitab Inc., State College, PA). Normality of the data was confirmed using Shapiro–Wilk test. Data are presented as the median with interquartile range ([IQR] 25th and 75th percentiles). Statistical difference between groups was determined using *t* test and Wilcoxon matched-pairs signed rank test. Correlation between groups was assessed using Pearson correlation coefficient test. Differences with a *p*-value less than 0.05 were considered statistically significant.

## Results

### Growth inhibitory activity

**Fig. 1:**
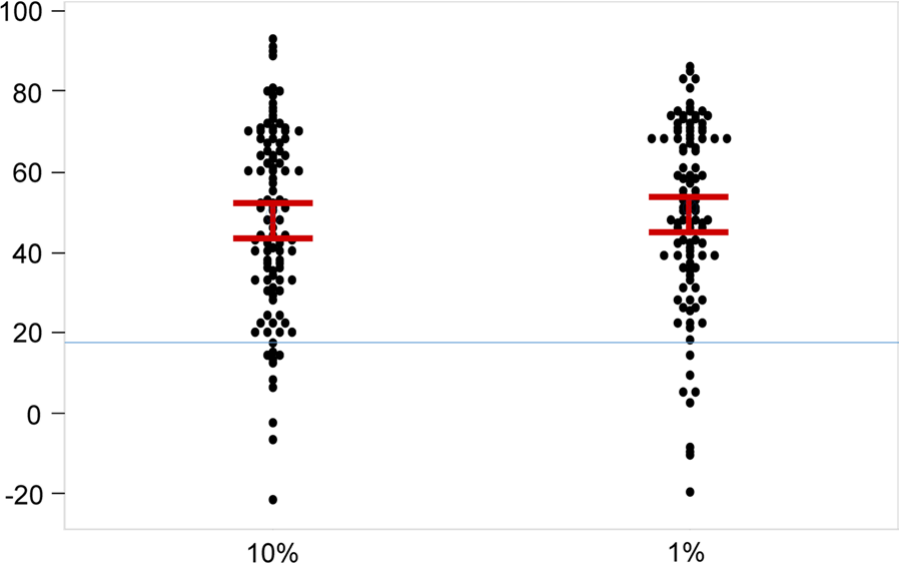
Anti-parasite activity in sera from study participants reaches saturation at a low testing concentration. Individual data plot represents in vitro growth inhibition of *P. falciparum* parasites (3D7) in the presence of 10% or 1% (v/v) serum concentration from immune patients. Circles indicate results for each study participant (n = 105). A serum pool consistent of six non-immune volunteers (indicated by blue line) was tested to determine background activity of the GIA assay. Median and 95% confidence interval are indicated for each experimental conditions

**Fig. 2:**
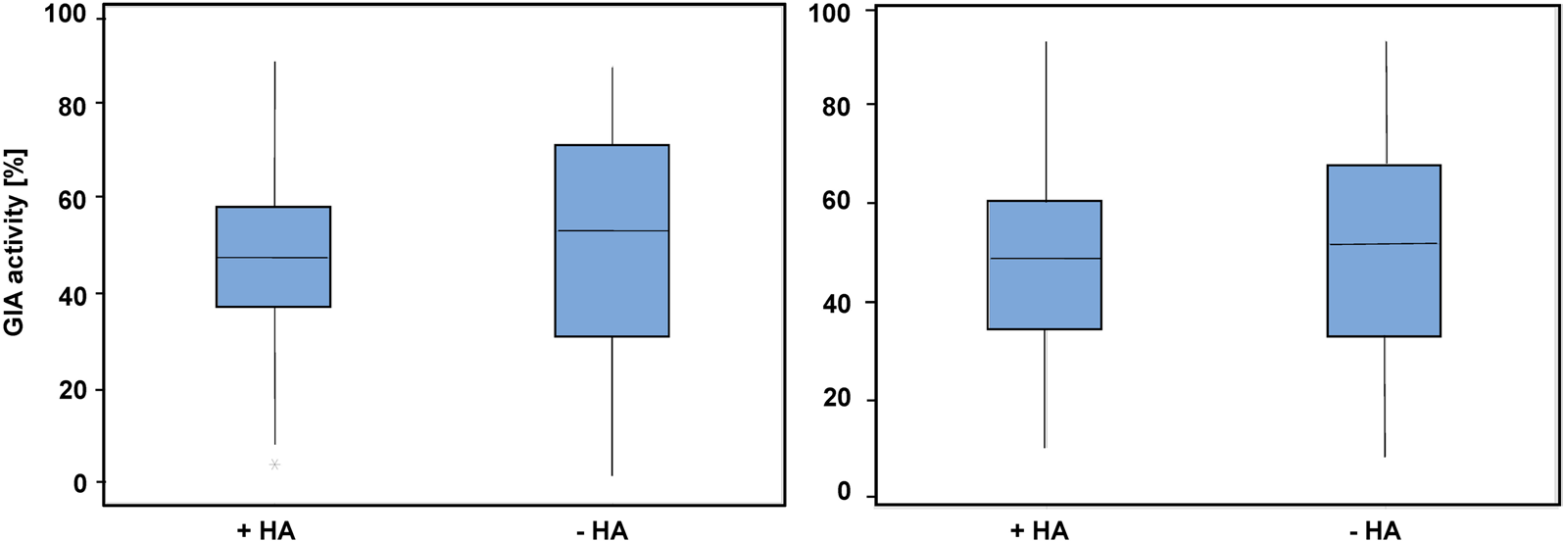
The contribution of complement to anti-parasite activity in sera is revealed at lower concentrations. Boxplot comparing GIA activity of Sera tested at 1% (left panel) (n = 105) and 10% (right panel) n = 105) with (+HA) and without (−HA) complement inactivation

The in vitro growth inhibition of *P. falciparum* in serum from the 105 immune study participants and non-immune were compared using Wilcoxon matched pair signed rank test. The percentage growth inhibition of *P. falciparum* at 10 and 1% serum concentration from the study participants was significantly higher than for the non-immune controls (*p* < 0.0001) (Fig. 1), indicating parasite growth inhibition was due to the immune status. There was no statistically significant difference in percentage growth inhibition for heat inactivated (+HA) serum compared to non-heat inactivated (−HA) serum at 10% serum concentration (*p* = 0.18). Conversely, the percentage growth inhibition was significantly higher for −HA serum than +HA serum at 1% serum concentration (*p* = 0.009) (Fig. 2). The results revealed the role of complement when the assay was performed at 1% serum concentrations where antibodies are at limiting concentrations.

### Age and GIA activity

**Fig. 3:**
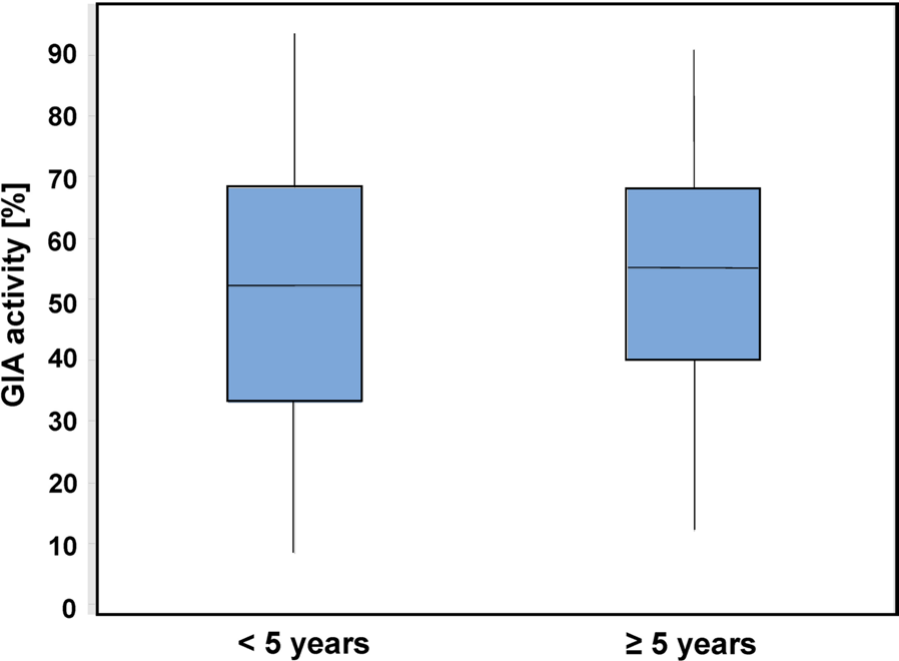
Age is not a confounding factor in the entire patient population. Boxplot showing impact of age < 5 years (n = 46) and ≥ 5 years (n = 59) on GIA in sera from symptomatic patients

To determine whether age or parasitaemia were confounding factors in GIA, the GIA data were stratified based on individuals younger than 5 years (< 5 years) or 5 years and older (≥ 5 years). There was no age-dependence of GIA activity nor significant correlation between GIA activity and parasite density (R^2^ = 0.03, p = 0.95 Pearson correlation coefficient) (Fig. 3).

### Impact of pre-existing immunity, GIA and treatment outcome

The main question of the present study was whether pre-existing immunity had an impact on the parasitological outcome after drug treatment and the kinetic of the response. The mean T_1/2_ for both study arms combined was 2.38 h (95% confidence interval [CI], 2.23–2.52), the median was 2.40 h (interquartile range [IQR], 1.95–2.80), and the range was 0.97–4.22 h. The cutoff values for stratifying into the two different groups have previously been described [19, 20]. Correlations between drug treatment outcome as determined by parasite clearance parameters (slope half-life [T_1/2_] and clearance rate [K]) and pre-existing GIA activity in sera were assessed (Table 2). The main observation when performing the statistical analyses was that stratifying the data based on the kinetic of the drug response revealed two factors, complement and age were associated with a faster parasite clearance and slope half-life. Non-heat inactivated sera from study participants younger than 5 years showed a significant negative correlation between GIA activity and slope half-life (R^2^ = 0.698, p = 0.025 Pearson correlation) and a significant positive correlation between GIA activity and parasite clearance (R^2^ = 0.604, p = 0.05 Pearson correlation). These correlations are lost when sera were heat-inactivated, clearly demonstrating the role of complement in clearing the parasite under drug treatment.

**Table 2:** Correlation between parasitological response to drugs and pre-existing GIA activity

Clearance kinetic ^a^	Age group	n subjects	T_1/2_ vs GIA^b^		K vs GIA^b^	
			−HA^c^	+ HA^c^	−HA	+ HA
< 2.02	< 5 years	9	R^2^ = − 0.698 p = 0.025^*^	R^2^ = − 0.413 p = 0.207	R^2^ = 0.604 p = 0.05^*^	R^2^ = 0.302 p = 0.367
	≥ 5 years	16	R^2^ = − 0.181 p = 0.518	R^2^ = − 0.042 p = 0.886	R^2^ = − 0.03 p = 0.915	R^2^ = − 0.043 p = 0.878
> 2.02	< 5 years	37	R^2^ = 0.21 p = 0.304	R^2^ = 0.193 p = 0.335	R^2^ = − 0.217 p = 0.208	R^2^ = − 0.158 p = 0.432
	≥ 5 years	43	R^2^ = − 0.017 p = 0.923	R^2^ = − 0.221 p = 0.217	R^2^ = 0.037 p = 0.837	R^2^ = 0.266 p = 0.2

^a^Stratification of study participants based on Slope half-life T1/2 ^17^ measured in hours

^b^Correlation expressed as Pearson correlation coefficient (R^2^); asterisk indicates statistical significance (p ≤ 0.05)

^c^GIA activity assessed in sera without (−HA) or with heat-inactivation (+HA)

## Discussion

The present study utilized samples from a TES study with a comprehensive immunological profile [20] and parasitological characterization. To complete the immune-parasitological profile, the functional activity of sera collected prior to drug treatment was assessed in a sensitive GIA assay [14]. To determine the contribution of innate factors such as complement, sera were tested with and without heat-inactivation. The initial analysis showed that the contribution of complement to the anti-parasite activity was only notable at low serum concentrations, where anti-malarial antibodies are likely under limiting concentrations. This confirms previous reports demonstrating the role of complement in GIA activity [7, 20].

The next step was to determine the impact of age on the GIA activity. Contrarily to a previous study [21], an age-dependent response in the functional activity was not displayed by the sera. This contradiction is likely because in the current study, population is comprised of participants that presented with malaria symptoms while other studies investigated the role of age in asymptomatic populations.

The key observation of this study was made when the data were stratified based on the drug kinetic response of the study participants [20]. This classification revealed that the parasitological response (i.e., parasite clearance rate and slope half-life) to drug was linked to GIA activity in non-heat-inactivated sera in patients under the age of five (Table 2). This finding suggests that the role of complement varies depending on the age of the study participant, corroborating previous studies that have demonstrated the importance of age in immune acquisition [22–24]. Previous assessment of the serological antibody profile did not find differences between the age groups [20] and, therefore, findings in this study points towards a greater role of innate factors in younger patients. The need for complement and complement fixing antibodies has been reported to be crucial for the protection against malaria clinical disease [7], supporting observation in this study that heat-inactivated serum has lower GIA activity. It does not preclude that there may be differences in the levels of malaria-specific IgM which has not been assessed. This is the main antibody isotype fixing complement and there may be differences in the distribution of malaria-specific IgM and IgG in the different age groups.

## Conclusion

In conclusion, the present study reveals differential relationships between adaptive immunity, innate immunity (mainly the role of complement), and response to drug treatment as function of age of the symptomatic study participants. Anti-parasitic activity (GIA) significantly correlated with T_1/2_ and K only in sera of younger symptomatic patients. This effect is dependent of intact (non-heat inactivated serum) indicating the importance of innate factors such as complement. These findings provide insight into improvement in effective use of ACT (dose adjustments) in relation to age in malaria endemic areas.

## Data Availability

All data generated or analysed during this study are included in this published article.

## Abbreviations

ACT: Artemisinin-based combination therapy
AL: Artemether-lumefantrine
AMA-1: Apical membrane antigen 1
ASMQ: Artesunate mefloquine
BEI: Biodefense and Emerging Infections Research Resources Repository
CMS: Complete media with serum
CNHR: Consortium for National Health Research
DEID: Department of Emerging and Infectious Diseases
DHA: Dihydroartemisinin
EIR: Entomological Inoculation Rates
GIA: Growth Inhibition Assay
−HA: Non-heat inactivated
+HA: Heat inactivated
K: Clearance rate
K/hour: Parasite clearance rate constant
KEMRI: Kenya Medical Research Institute
LU: Lumefantrine
MSP-1: Merozoite surface protein 1
NAI: Naturally acquired immunity
PCE: Parasite clearance estimator
PCR: Polymerase chain reaction
RBC: Red blood cell
RFUs: Relative fluorescence units
sSA: sub-Saharan Africa
TCM: Tissue culture media
TESs: Therapeutic efficacy studies
T_1/2_: Slope half-life
USAMRD-A: United States Army Medical Research Directorate-Africa
WBCs: White blood cells
WHO: World Health Organization
WRAIR: Walter Reed Army Institute of Research
WRP: Walter Reed Project
